# Phenotypic Variation Is Almost Entirely Independent of the Host-Pathogen Relationship in Clinical Isolates of *S*. *aureus*

**DOI:** 10.1371/journal.pone.0129670

**Published:** 2015-06-22

**Authors:** Adrian D. Land, Patrick Hogan, Stephanie Fritz, Petra Anne Levin

**Affiliations:** 1 Department of Biology, Washington University in Saint Louis, Saint Louis, Missouri, United States of America; 2 Department of Pediatrics, Washington University School of Medicine, St Louis, Missouri, United States of America; University of Groningen, Groningen Institute for Biomolecular Sciences and Biotechnology, NETHERLANDS

## Abstract

**Background:**

A key feature of *Staphylococcus aureus* biology is its ability to switch from an apparently benign colonizer of ~30% of the population to a cutaneous pathogen, to a deadly invasive pathogen. Little is known about the mechanisms driving this transition or the propensity of different *S*. *aureus* strains to engender different types of host-pathogen interactions. At the same time, significant weight has been given to the role of specific *in vitro* phenotypes in *S*. *aureus* virulence. Biofilm formation, hemolysis and pigment formation have all been associated with virulence in mice.

**Design:**

To determine if there is a correlation between *in vitro* phenotype and the three types of host-pathogen relationships commonly exhibited by *S*. *aureus* in the context of its natural human host, we assayed 300 clinical isolates for phenotypes implicated in virulence including hemolysis, sensitivity to autolysis, and biofilm formation. For comparative purposes, we also assayed phenotype in 9 domesticated *S*. *aureus* strains routinely used for analysis of virulence determinants in laboratory settings.

**Results:**

Strikingly, the clinical strains exhibited significant phenotypic uniformity in each of the assays evaluated in this study. One exception was a small, but significant, correlation between an increased propensity for biofilm formation and isolation from skin and soft tissue infections (SSTIs). In contrast, we observed a high degree of phenotypic variation between common laboratory strains that exhibit virulence in mouse models. These data suggest the existence of significant evolutionary pressure on the *S*. *aureus* genome and highlight a role for host factors as a strong determinant of the host-pathogen relationship. In addition, the high degree of variation between laboratory strains emphasizes the need for caution when applying data obtained in one lab strain to the analysis of another.

## Introduction

*Staphylococcus aureus* is an important gram-positive pathogen that colonizes the skin and mucosal membranes of 30% of the population [1]. While generally associated with minor skin infections, *S*. *aureus* can also be associated with more invasive disease. *S*. *aureus* is a highly successful pathogen in part because it has numerous functionally redundant virulence factors [2,3]. *S*. *aureus’* ability to acquire antibiotic resistance without a significant impact on growth or virulence has made it a serious public health concern [4,5].

A common cause of nosocomial infections, *S*. *aureus* can become epidemic within hospitals where it is easily passed between patients and healthcare providers. Up to 75% of newborns are carriers of *S*. *aureus* by the time they are 5 days old [6]. Methicillin-resistant *S*. *aureus* (MRSA) was historically limited to patients with significant healthcare exposure, and these healthcare-associated MRSA strains are particularly problematic due to the limited number of antibiotics available for treatment. Recently evolved community-associated MRSA (CA-MRSA) infections are an emerging public health problem. CA-MRSA outbreaks are generally associated with superficial skin and soft tissue infections (SSTI), however there have been cases of invasive and sometimes deadly diseases that account for ~20,000 deaths per year. The emergence of CA-MRSA and the pre-existing prevalence of MRSA in hospital settings have made *S*. *aureus* the most deadly infectious agent in the United States [7].

Much of what we know about the physiology and virulence of *S*. *aureus* has been elucidated through genetic disruption of assayable phenotypes in one of several domesticated laboratory strains and subsequent analysis of virulence in mouse models. Although many such studies have speculated that particular virulence determinants may be associated with a particular host-pathogen interaction, we know little about the factors that underlie these interactions in clinical settings.

Here we present comparative phenotypic data from the analysis of 9 strains of *S*. *aureus* commonly used in virulence studies as well as 300 CA-MRSA clinical isolates that have been subject to minimal passaging. We utilized a number of laboratory assays that have been correlated with staphylococcal virulence in mouse models to examine the extent of phenotypic variation in this collection of “wild type” *S*. *aureus* strains. Our observations reinforce the need to use caution when developing models for *S*. *aureus* virulence mechanisms using these profoundly different strains.

## Experimental Design

To identify *S*. *aureus* determinants correlated with three types of host-pathogen interaction: commensal, SSTI, and invasive infection, we subjected 9 domesticated “wild type” laboratory strains and 300 clinical isolates of *S*. *aureus* to systematic phenotypic analysis. The nine, highly passaged, laboratory strains included four laboratory strains commonly used for the study of *S*. *aureus* virulence (NCTC 8325, SH1000, Newman, UAMS1), two health care acquired methicillin resistant strains (N315 and Mu50), and three community acquired methicillin resistant strains (USA400 MW2, USA 300 TCH1516, and USA300 FPR3757). The three hundred clinical isolates were obtained between 2008 and 2011 from patients at St. Louis Children’s Hospital (SLCH) and subjected to only minimal passaging.

### Domesticated laboratory strains

NCTC 8325 is the progenitor strain of the 8325 lineage and one of the first reported sequenced genomes of *S*. *aureus* [8]. Members of this lineage are often used as model organisms in many facets of laboratory research [9,10]. SH1000 is an NCTC 8325 derivative that has been cured of its 3 prophages and is frequently employed for biofilm studies [9]. Newman, was isolated from a human infection and is described as having a “robust” virulent phenotype [11]. Finally, UAMS1 was isolated from a biopsy of an osteomyelitis patient and is frequently employed in studies of musculoskeletal infections [12]. All of these strains have been passaged for many years. Newman and NCTC8325 in particular were isolated in 1943 and 1952 respectively [8,11].

#### HA-MRSA strains

In addition to methicillin-sensitive *S*. *aureus* (MSSA) strains, we also compared two methicillin-resistant healthcare associated-MRSA strains, N315 and Mu50 (Table 1). MRSA origin strains N315 and Mu50 were isolated from patient infections in 1982 and 1997 respectively [13]. The Mu50 strain shows intermediate resistance to the glycopeptide antibiotic vancomycin, which is commonly used in the treatment of serious MRSA infections. Intermediate susceptibility to vancomycin (VISA) is associated with persistent *S*. *aureus* infections, and results from a variety of mutations in cellular processes including cell wall synthesis, transport, toxin synthesis, antibiotic resistance, and metabolism. A key feature of VISA strains is a reduced growth rate and corresponding small colony phenotype associated with aberrant cell wall turnover and altered metabolic function [14].

**Table 1:** *S*. *aureus* strains.

Strain	Description
NCTC 8325	Standard laboratory strain. Propagating strains for phage 47.
SH1000	Standard laboratory strain. Man made derivative of NCTC 8325 that has been cured of prophages with functional *rsbU*.
Newman	Standard laboratory strain. Isolated from human infection and used extensively in animal models of staphylococcal disease.
UAMS1	Standard laboratory strain. Isolated from biopsy of osteomyelitis patient. Used in virulent studies of staphylococcal disease.
Mu50	Vancomycin-intermediate strain isolated from wound of infected patient.
N315	Methicillin-resistant strain isolated from respiratory tract of infected patient.
TCH1516	Strain type USA300. CA-MRSA isolated from otherwise healthy patient. Used in virulence studies in mouse model systems.
FPR3757	Strain type USA300. CA-MRSA isolated from wound of HIV positive intravenous drug user.
MW2	Strain type USA400. CA-MRSA strain isolated from blood of infected patient.

#### CA-MRSA strains

Three CA-MRSA strains were also included in the study: USA400 MW2, USA 300 TCH1516, and USA300 FPR3757 (Table 1). USA400 MW2, isolated in 1998, caused the first recorded fatalities attributed to a MRSA infection acquired outside the hospital [15]. USA300 FPR3757 was isolated in 2002, from the wound infection of a patient with a SSTI, while USA300 TCH1516 isolated in 2001, was taken from the blood of a patient suffering from severe sepsis [16,17]. Comparisons of the whole genome sequence of these 2 strains have revealed just 34 non-synonymous SNPs in proteins of various functions [17]. However no previous data exists on phenotypic differences associated with these changes other than antibiotic susceptibility profiles.

### Clinical Isolates

Clinical isolates were collected from various body sites (e.g. nares, soft tissue, blood, and bones) and following minimal manipulation to select for single colonies and test for antibiotic susceptibility, frozen and stored. These strains represent 3 different classes of *S*. *aureus* host-pathogen interactions, given the tissues from which they were collected (39%-colonization, 50%-SSTI, 11%-invasive). Of the 300 clinical isolates examined, 95% were sequence typed as being USA300/ST8 (multilocus sequence type 8, clonal complex 8, staphylococcal cassette chromosome *mec* type IV), the predominant cause of community acquired MRSA in the United States [18]. Note that the number of invasive isolates used in this study is an overrepresentation relative to average patient intake at SLCH.

## Results

### Pigment formation is highly variable in reference strains

Differences in growth rate, colony size, and colony pigment have been associated with *S*. *aureus* virulence and antibiotic sensitivity [19–22]. A prime example is *S*. *aureus* strains with an intermediate sensitivity to vancomycin, which is known to have a growth defect in rich medium [13,23,24]. Similarly, pigment color can be a strong indicator of *S*. *aureus* environmental phenotype. The golden color of *S*. *aureus* is associated with the production of the pigment staphyloxanthin, which is involved in host evasion [6]. Unpigmented strains of *S*. *aureus* succumb to the immune system more rapidly than pigmented strains [20,21,25].

To identify physiological costs associated with different adaptive mutations we evaluated the growth properties of the 9 domesticated strains of *S*. *aureus* in Tryptic Soy Broth (TSB). We observed *S*. *aureus* isolates relative growth rates, cell density, and survival potential in stationary phase. Single colonies of *S*. *aureus* were inoculated into TSB and grown overnight at 37°C with shaking. Following overnight incubation, cultures are diluted back to an OD_600_ of ~0.05 and grown at 37°C with shaking. Optical density was measured every 30 minutes. We also observed the colony morphology of each of the clinical isolates and control strains on solid Tryptic Soy Agar (TSA) media to identify changes in pigment formation.

**Fig 1:**
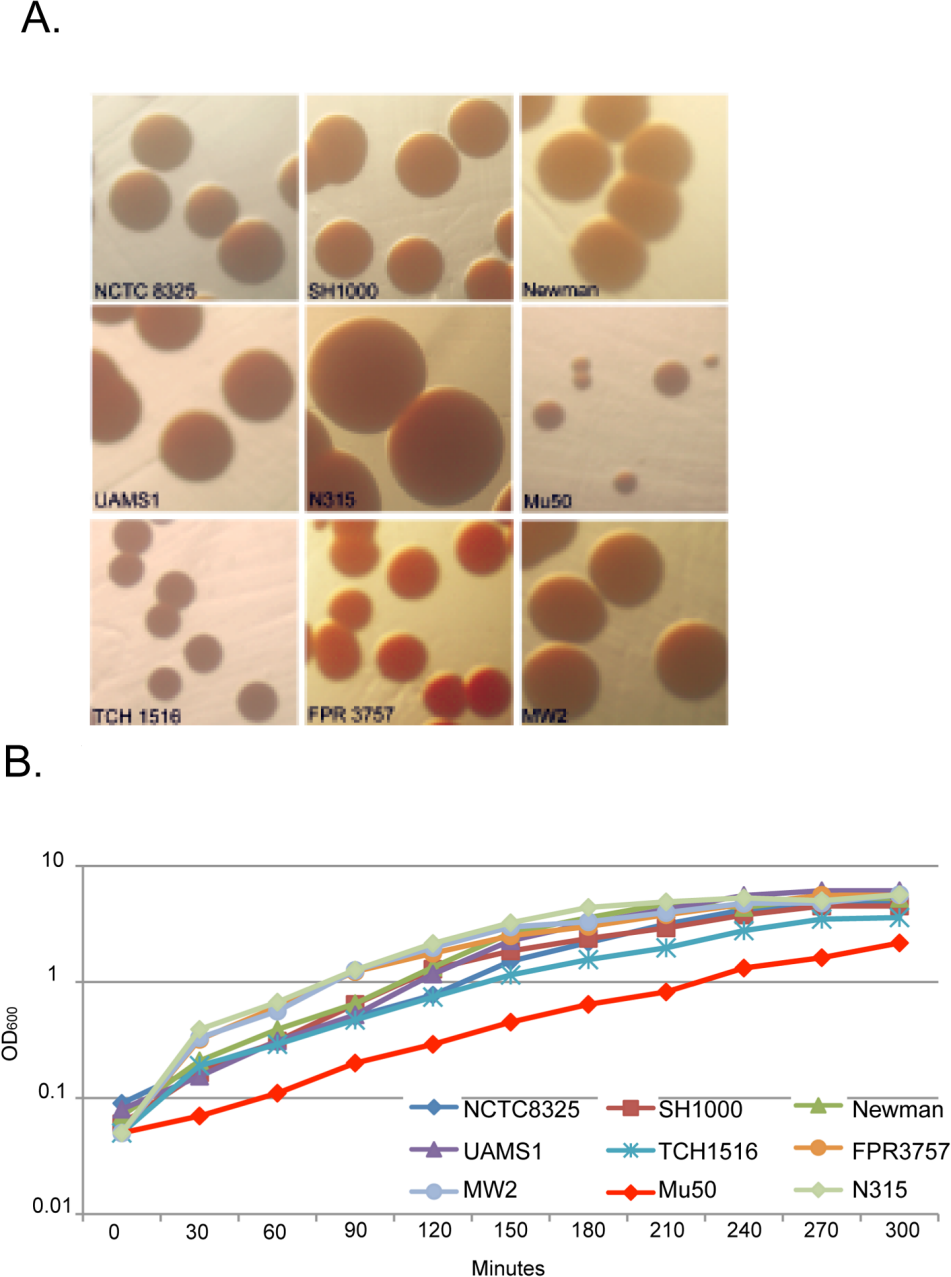
Colony morphology and growth rate variability in reference strains of *S*. *aureus*. (A) Domesticated strains of *S*. *aureus* were streaked onto TSA plates and grown overnight at 37°C. Following incubation images were observed for differences in color and size. (B) We observed *S*. *aureus* isolates relative growth rates, cell density, and survival potential in stationary phase. Single colonies of *S*. *aureus* were inoculated into TSB and grown overnight at 37°C with shaking. Following overnight incubation cultures are diluted back to an OD600 of ~0.05 grown at 37°C with shaking. Optical density was measured every 30 minutes.

Comparisons of the growth rates of each domesticated staphylococcal strain revealed very few differences in cell densities and mass doubling times. As expected the VISA strain Mu50 grew substantially slower in Tryptic Soy Broth (TSB) when compared to the other reference strains (Fig 1B). This growth property is common amongst VISA isolates and is thought to be due to defects in cell wall biosynthesis [14]. Also Mu50, plated on TSA plates appeared smaller by visual analysis, than the related N315 strain and the other laboratory strains. Observations of the domesticated laboratory strains plated on TSB agar revealed a distinct difference in the pigmentation of SH1000 and its progenitor strain NCTC 8325 (Fig 1A). Comparisons of the two USA300 strains directly exhibited differences in both growth rates and yields. USA300 TCH1516 grows slower in TSB media than its subclone USA300 FPR3757 (Fig 1B). There were also morphological distinctions when comparing the three USA300 strains. TCH1516 formed small, unpigmented colonies on TSA plates, while FPR3757 and MW2 both formed larger golden colonies under identical growth conditions (Fig 1A).

### Sensitivity to TX100 Autolysis varies as much as 10-fold in reference strains

Bacterial autolysis is the self-digestion of a cell through the actions of its own peptidoglycan hydrolases. Because of the lethality associated with compromised cell wall integrity, the actions of these enzymes are tightly regulated. Perturbations of autolysis have been associated with defects in cell wall synthesis or cell wall turnover, antibiotic resistance, and virulence [26,27].

We tested the autolytic activity of each of the domesticated strains of *S*. *aureus* by exposing them to low concentrations of Triton X-100 (TX-100). Single colonies were grown as described above. Upon reaching stationary phase, cultures are chilled on ice to halt growth. Chilled cultures are then centrifuged at 15,000rpm and re-suspended in autolysis buffer containing TX-100. Sensitivity to TX-100 autolysis was reflected by a decrease in optical density upon exposure to TX-100.

**Fig 2:**
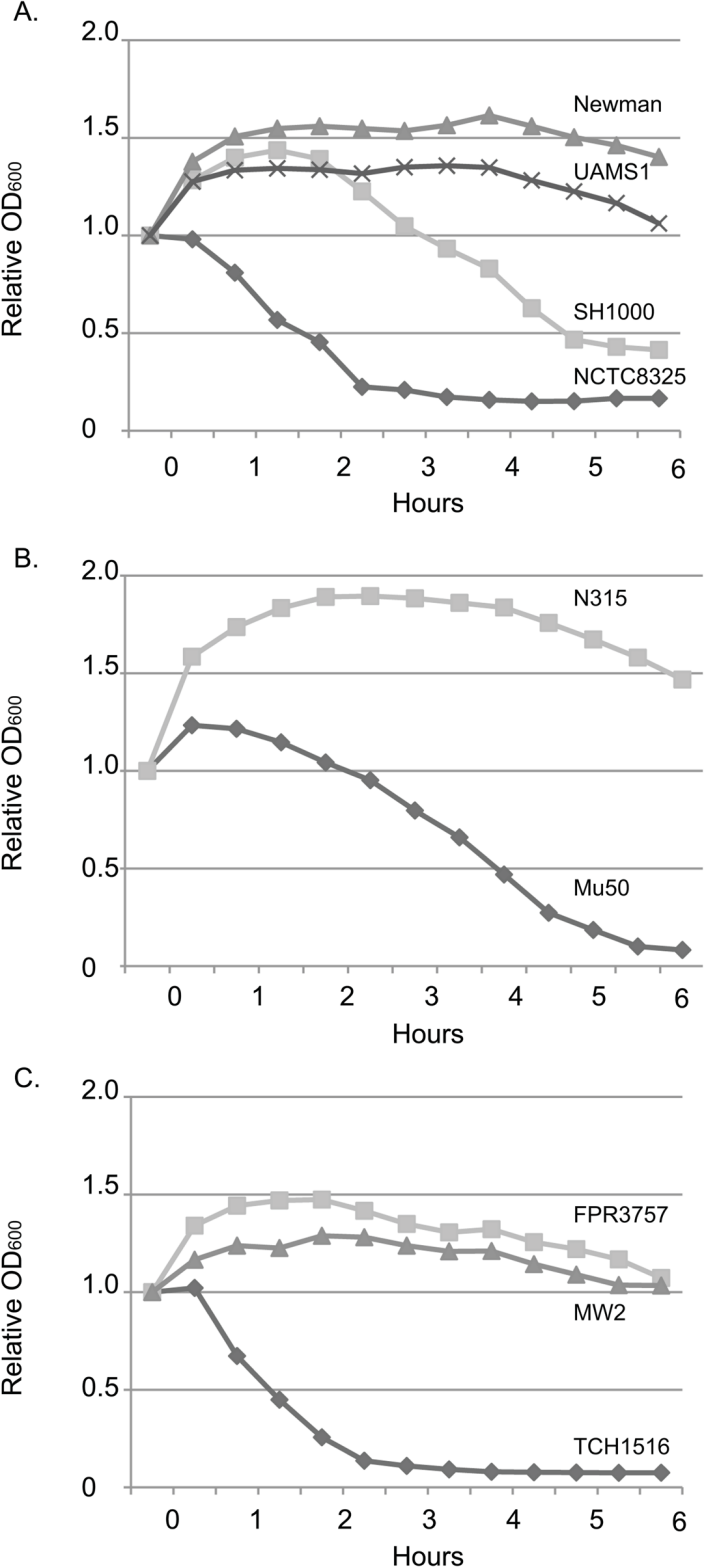
Sensitivity to autolysis varies <10 fold in reference strains. We tested the autolytic activity of each reference strain of *S*. *aureus* by exposing them to low concentrations of Triton X-100. Sensitivity to autolysis was reflected by a decrease in the OD_600_ upon exposure to TX-100. There was significant variability in sensitivity amongst the (A) Laboratory strains (B) HA-MRSA strains and (C) CA-MRSA strains of *S*. *aureus* observed in this assay.

The 9 domesticated strains exhibited tremendous variability in their autolytic activity following exposure to TX-100. NCTC8325 and Mu50 were highly sensitive to autolysis by TX-100, relative to related strains N315 and SH1000 respectively (Fig 2A and 2B). The OD_600_ of both NCTC8325 and Mu50 decreased rapidly two hours after exposure to the detergent (Fig 2A and 2B). This result was consistent with previous observations that Mu50 loses viability rapidly [28]. Strains Newman, N315, and UAMS1 were resistant to autolysis by TX-100 at the concentrations used in this assay. The CA-MRSA strains also exhibited a high degree of divergence with regard to autolytic susceptibility. Strain TCH1516 was highly susceptible (~10-fold) to autolysis by TX-100 when compared to its counterpart FPR3757 (Fig 2C), suggesting compromised cell wall or cell membrane integrity.

### The laboratory strains Newman and UAMS1, together with slow growing HA and CA-MRSA strains exhibit poor biofilm formation

Biofilm formation is thought to play a key role in *S*. *aureus* virulence. Biofilms have been implicated in invasive *S*. *aureus* infections and may be particularly important for the first step in pathogenesis, colonization [29,30]. Biofilms protect the bacteria from the host immune system increasing nonspecific antibiotic resistance [29,30]. *S*. *aureus* biofilm formation on indwelling medical devices has been associated with nosocomial infections including pneumonia [29–31]

**Fig 3:**
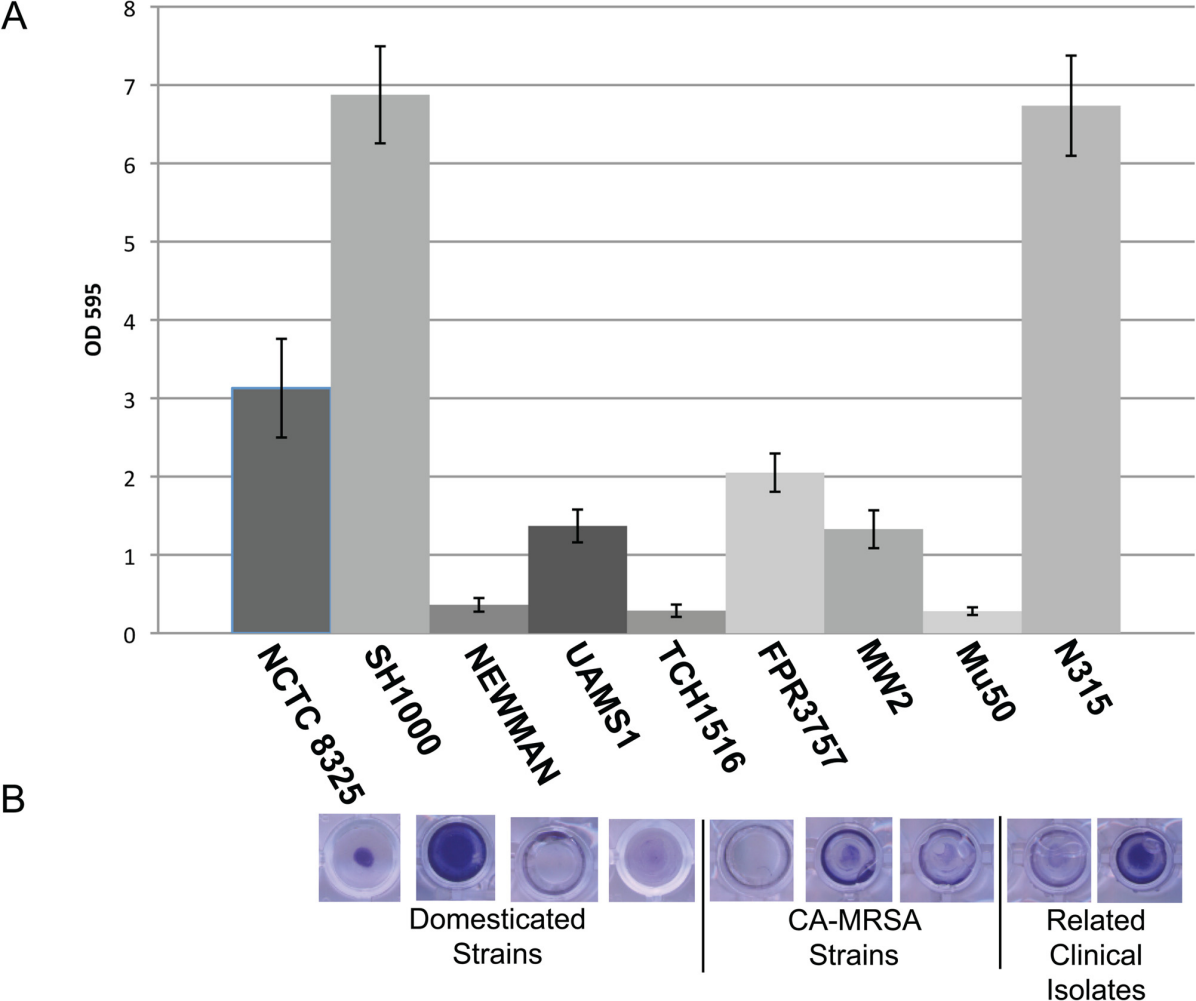
Laboratory strains Newman and UAMS1, together with slow growing HA and CA-MRSA strains exhibit poor biofilm formation. To determine the relative ability of *S*. *aureus* to form biofilms we used a standard 96-well plate assay on each of our staphylococcal strains [70,71]. (A) Single colonies of *S*. *aureus* were grown overnight in TSB + glucose. Cultures were backdiluted to an OD_600_ of 0.005 in TSB + glucose and 100ul aliquots of each strain are plated in triplicate in 96 well microtiter plates. Microtiter plates were grown statically at 37°C for 24hrs. Unattached cells are discarded and the remaining attached cells are then stained with crystal violet. Results depicted here represent the mean and standard deviation of 12 replicates of each reference strain type (B) Representative image of the crystal violet staining of microtiter plates for each reference strain. Images are taken from above.

To determine the relative ability of the domesticated *S*. *aureus* strains to form biofilms we used a standard 96-well plate assay on each of our staphylococcal strains (Materials and Methods). Single colonies of *S*. *aureus* were grown overnight in TSB + glucose. Cultures were backdiluted to an OD_600_ of 0.005 in TSB + glucose and 100ul aliquots of each strain are plated in triplicate in 96 well microtiter plates. Microtiter plates were grown statically at 37°C for 24hrs. The domesticated strains tested in the biofilm assays exhibited a high degree of variation (Fig 3).

SH1000, the standard strain for biofilm related studies, exhibited consistently strong biofilm formation, >5-fold higher than Newman and UAMS1. Strain N315 also formed robust biofilms near the density of SH1000, however closely related Mu50 is a poor biofilm forming strain. Neither of the CA-MRSA strains MW2, TCH1516 nor FPR3757 were strong biofilm formers (Fig 3). We speculate that slow growth is a major contributor to the poor biofilm forming ability exhibited by Mu50 and TCH1516.

### Hemolytic activity differs significantly between closely related laboratory, CA-MRSA and HA-MRSA strains

Host cell lysis is a key feature of a broad range of pathogens. Lysis is a means to obtain nutrients such as iron from the host cells [32,33]. Absence of hemolysis in *S*. *aureus* has been correlated with the down-regulation of either α or β-hemolysins, or mutations in the *agr* quorum sensing system [3,33–36]. Hemolysis is central to *S*. *aureus* ability to invade, colonize and obtain nutrients, particularly iron. Defects in hemolytic ability are associated with decreased virulence in mouse model systems [3,33–36]. A previous study done by *Malachowa et al* reported significant differences in the transcriptional profiles of *S*. *aureus* strains grown in blood compared to TSB [37]. The list of blood dependent up-regulated genes included numerous cytolytic toxins including *hla*, *hld*, and *hlgABC* [37].

To assess hemolytic activity, single colonies of *S*. *aureus* were grown up to exponential phase in TSB, then back-diluted to an OD_600_ of ~0.05. Diluted cultures are then spotted on TSA plates supplemented with 5% sheep’s blood and incubated for 48hrs. Following incubation the “zones of hemolytic clearing” were measured and recorded.

**Fig 4:**
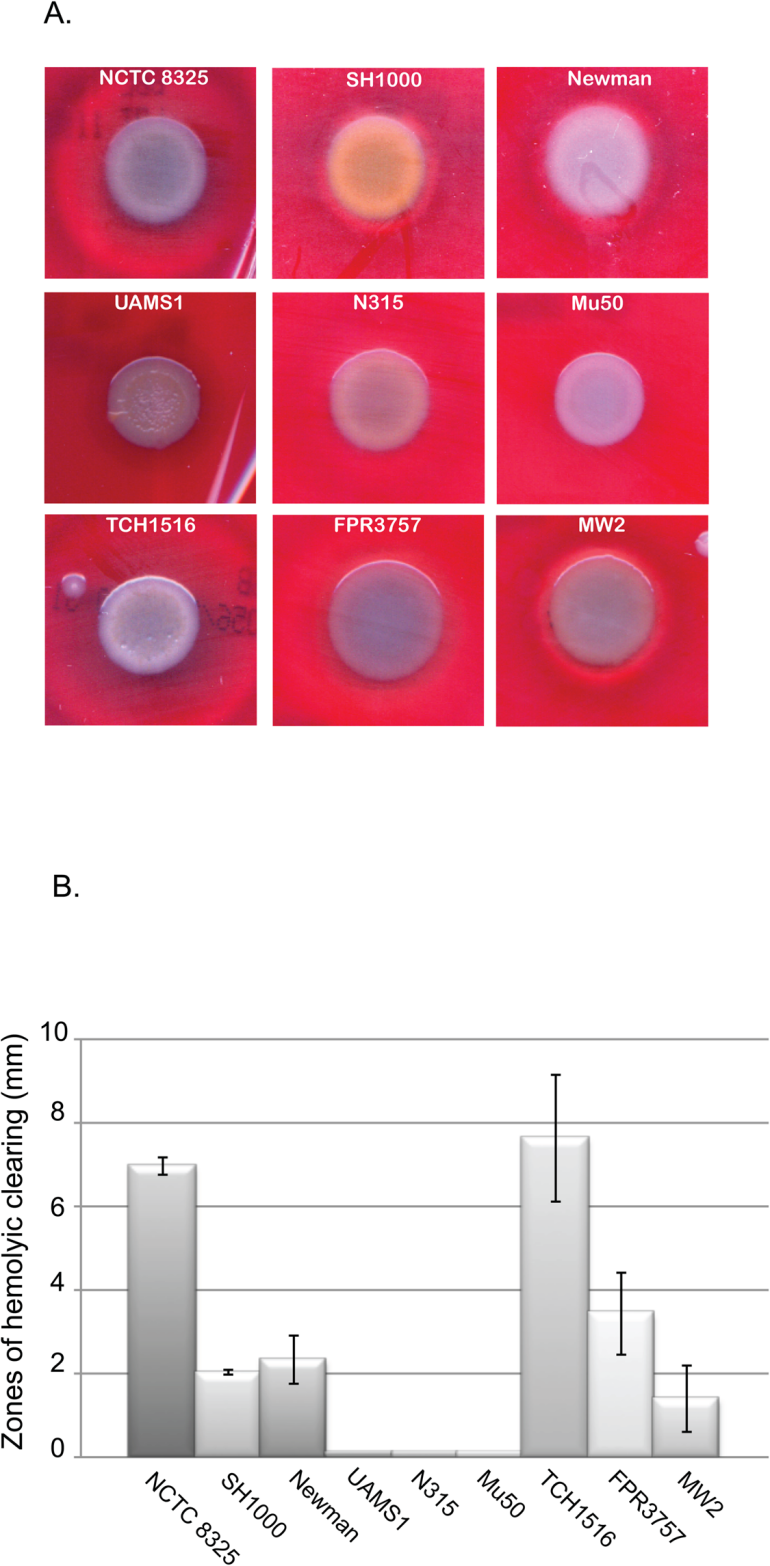
Hemolytic activity differs significantly between closely related laboratory, CA-MRSA and HA-MRSA strains. (A) Hemolytic activity was assessed by growing single colonies of *S*. *aureus* up to exponential phase in TSB, then back-diluted to an OD_600_ of ~0.05. Diluted cultures are then spotted on TSB agar plates supplemented with 5% sheep’s blood and incubated for 48hrs. Following incubation the “zones of hemolytic clearing” are measured and recorded. (B) Graphical representation of the “zones of hemolytic clearing” observed in reference strains. Data shown is average of 3 biological replicates. Error bars represent standard deviation.

There was significant variation of hemolytic activity amongst the domesticated strains of *S*. *aureus* including the closely related strains, NCTC 8325 and SH1000. A large zone of hemolysis was observed for NCTC 8325 that was approximately three times the size of that observed for SH1000 (Fig 4B). Three strains, UAMS1, N315, and Mu50 showed no hemolytic activity in this assay. The three CA-MRSA strains also exhibited a wide range of variation with regard to hemolytic activity, with zones of clearing in TCH1516, FPR3757, and MW2 showed 9mm, 4mm and 1mm zones respectively, indicating a significant difference in each strain’s ability to lyse sheep red blood cells (Fig 4A and 4B).

### The hyperhemolytic CA-MRSA strain TCH1516 is dependent on exogenous heme for growth

In the course of this investigation we noticed that USA300 TCH1516 exhibited extremely poor growth on TSA but growth was significantly improved on TSA + 5% sheep’s blood. This is in contrast to the other reference strains, which grew equivalently on TSA media in presence and absence of 5% sheep’s blood. Even Mu50, the VISA strain, exhibited equivalently poor growth regardless of medium.

In light of its enhanced growth on sheep’s blood, we speculated that the root cause of the TCH1516 growth defect was a heme deficiency. Since TCH1516 was isolated from human blood, it is possible that it lost the ability to synthesize heme during its transition from commensal to invasive infection. To test this possibility, we compared the growth of TCH1516, FPR3757, and Newman on TSA, TSA supplemented with human hemoglobin, and TSA + 5% sheep’s blood. Briefly, TCH1516, FPR3757, and Newman were grown up to exponential (OD ~0.5), backdiluted to OD_600_ of ~0.05, and serially diluted (10^−1^–10^−5^) on either TSA plates or TSA plates supplemented with human hemoglobin or sheep’s blood.

**Fig 5:**
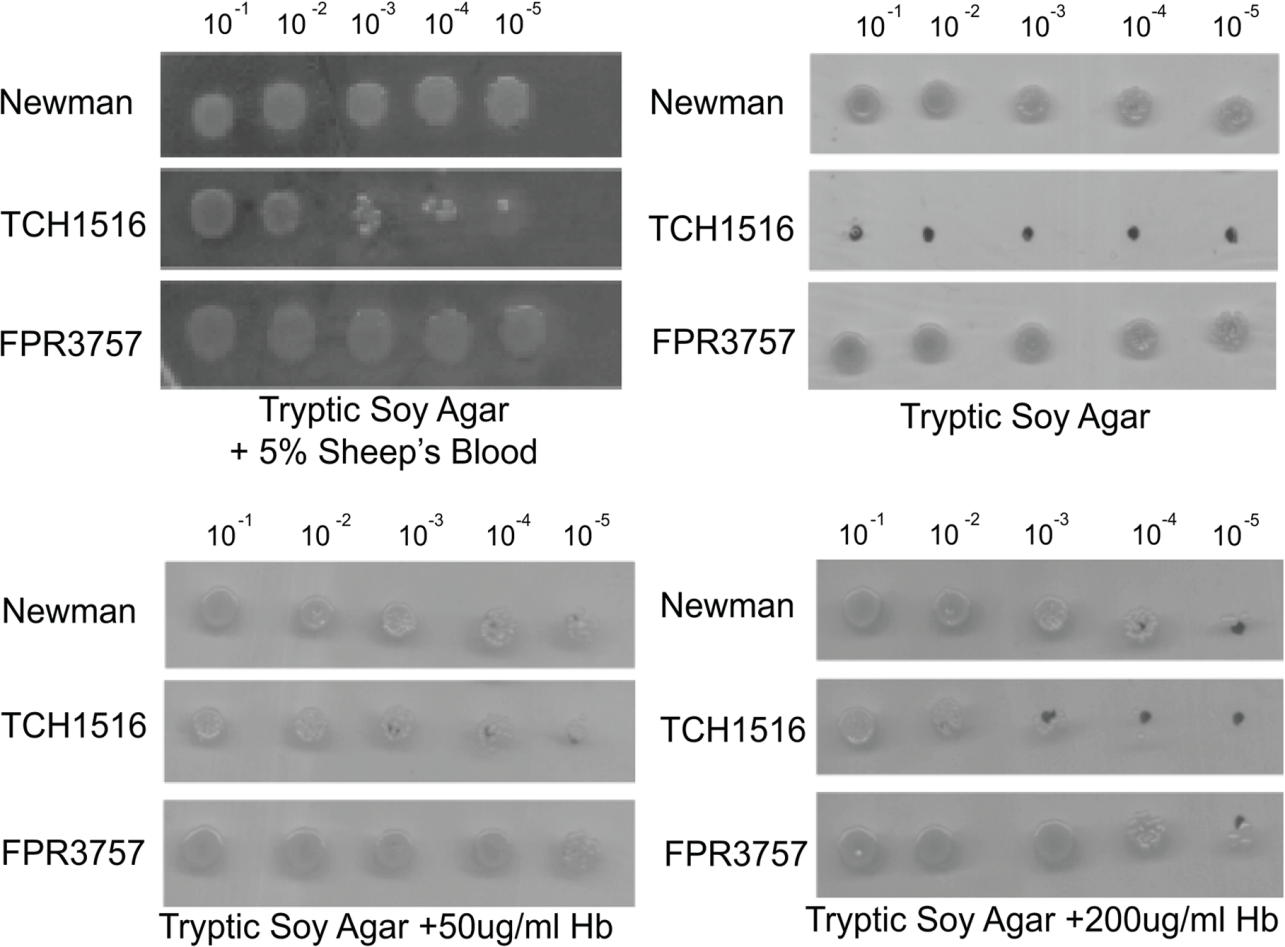
The hyperhemolytic CA-MRSA strain TCH1516 is hemoglobin-dependent for growth. TCH1516, FPR3757 and Newman were grown up to exponential (OD ~0.5) backdiluted to OD_600_ of ~0.05, and serially diluted (10^−1^–10^−5^) on either TSA plates or TSA plates supplemented with human hemoglobin or sheep’s blood.

Consistent with observations of heme deficient strains, supplementing the solid medium with 50ug/ml or 200ug/ml of hemoglobin or 5% sheep’s blood substantially improved the growth of the USA300-TCH1516 strain (Fig 5) but had little impact on the relatively robust growth of Newman and FPR3757. Based on these findings, we speculate that USA300 TCH1516 is a heme auxotroph. Consistent with this idea, sequence analysis revealed allelic differences in the heme biosynthetic pathway of TCH1516 [17]. We will address the significance of these SNPs in discussion.

### Clinical Isolates of USA300 demonstrate phenotypic homogeneity regardless of their origin

We assayed ~300 clinical isolates of *S*. *aureus* for three different phenotypes (pigment formation, hemolysis, acetate utilization) that have been implicated in virulence to examine if there is a correlation between laboratory phenotype and a given type of host-pathogen interaction.

Our collection of clinical strains includes isolates from the three clinical entities; skin and soft tissue infections, invasive infections, and sites of colonization (commensal). Multidrug-resistant and multidrug-susceptible strains are included in the collection; however there is no correlation between clinical presentation and multidrug resistance [3]. The majority, (285/300; 95%) of these isolates have been classified as ST8 (USA300), a community-acquired *S*. *aureus* variant that can acquire multidrug resistance.

The 300 clinical isolates were considerably more homogeneous with regard to colony morphology, hemolysis activity, and acetate utilization than the laboratory strains. While we saw some bias in biofilm forming ability, particularly with regard to isolates sampled from SSTIs, this bias was quite modest. Also we did not observe any significant differences between the ST8 strains and the 15 “other” strain types. Below we detail our findings for each of the three phenotypic assays.

#### Colony Pigment

Colony pigment was assessed on TSA plates after 24-hour incubation at 37°C as described above for laboratory strains. An overwhelming majority (96%) of the strains examined were the deep gold characteristic of wild type *S*. *aureus*. Surprisingly, invasive strains were more likely to have a white colony pigment (16%) than colonizing or SSTI strains (both 3%; p = 0.002) (Table 2). Down-regulation of the staphyloxanthin pigment has been associated with evasion of the human immune system [20,21,25]. The ability of many CA-MRSA strains to escape the host immune response undoubtedly enhances their ability to become an infectious agent.

**Table 2:** Statistical analysis of phenotype variation in clinical isolates.

	Total	Colonization	SSTI	Invasive	
	N = 300 (%)	N = 117 (%)	N = 151 (%)	N = 32 (%)	p-value
Colony Morphology					
Gold	288 (96)	114 (97.4)	147 (97.4)	27 (84.4)	0.002
White	12 (4)	3 (2.6)	4 (2.6)	5 (15.6)	
Hemolytic Activity					
Low/none	13 (4.3)	8 (6.8)	3 (2)	2 (6.3)	0.186
Intermediate	272 (90.7)	101 (86.3)	143 (94.7)	28 (87.5)	
High	15 (5.0)	8 (6.8)	5 (3.3)	2 (6.3)	
Acetate Utilization					
Positive	287 (95.7)	112 (95.7)	147 (97.4)	28 (87.5)	0.045
Negative	13 (4.3)	3 (4.3)	4 (2.6)	4 (12.5)	

#### Hemolytic activity

The hemolytic activity of all 300 clinical isolates was evaluated based on the zone of clearing around single colonies plated on TSA + 5% sheep’s blood, as described above. TCH1516 (high hemolysis >5mm), FPR3757 (intermediate hemolysis between 1-5mm) and UAMS1 (no hemolysis <1mm) were used as reference strains. Plates were observed following 24hr incubation at 37°C. Each colony was examined for altered hemolytic phenotypes. A large majority (91%) of the observed strains had an intermediate phenotype, similar to FPR3757 (Fig 4). Colonizing (14%) and invasive (13%) strains showed a higher incidence of altered hemolytic activity than SSTI (5%) strains, though this difference did not reach statistical significance (Table 2).

#### Acetate utilization

The TCA cycle in *S*. *aureus* is activated in the stationary growth phase as glucose levels decrease and acetate increases [38]. Inactivation of the TCA cycle has been shown to disrupt many factors related to host-pathogen interactions including virulence, survival, and persistence [28,38–42]. In *S*. *aureus* the ability to catabolize acetate in the post-exponential growth phase has been correlated to increased virulence factor production [38].The inability to generate energy through the oxidation of acetate is associated with resistance to oxidative stress and reduced susceptibility to antibiotics [41]. We evaluated the variation of acetate catabolism in the stationary growth phase of 300 clinical isolates of *S*. *aureus*.

Acetate utilization was evaluated in cells cultured in TSB media and phenol red as pH indicator as described by Greg Somerville (Personal Communication). Acetate catabolism is evidenced by a color change from yellow to pink after overnight (~18 hours) of growth in TSB + indicator medium at 37°C. An acidic pH is indicative of an inability to utilize acetate.

Consistent with the idea that the TCA cycle has an important role in *S*. *aureus* physiology, the overwhelming majority of the 300 clinical isolates we evaluated were positive for acetate catabolism (96%) as judged by visual analysis. While invasive strains (88%) were statistically (p = 0.045) less likely to efficiently utilize acetate than colonizing (96%) or SSTI (97%) strains, the physiological basis for this difference is not readily apparent (Table 2).

#### Biofilm formation

As discussed in the previous section, biofilm formation has been strongly implicated in virulence in laboratory strains of *S*. *aureus*, particularly SH1000 [29,30,43,44]. In particular, it has been suggested that the ability to form biofilms enhances *S*. *aureus* resistance to disinfectant and antibiotic treatment, as well as to colonize abiotic surfaces and human skin. The latter is particularly important because of the potential to become a reservoir for infections in both hospital and community settings [31].

To determine if differences in biofilm forming ability correlate with different types of host-pathogen relationships, we assessed relative biofilm formation in a microtiter plate assay in a subset of the 300 *S*. *aureus* clinical isolates. These 96 strains included the entirety of invasive-infecting strains (32 total), and 32 randomly selected strains from SSTI and colonization strains respectively.

**Fig 6:**
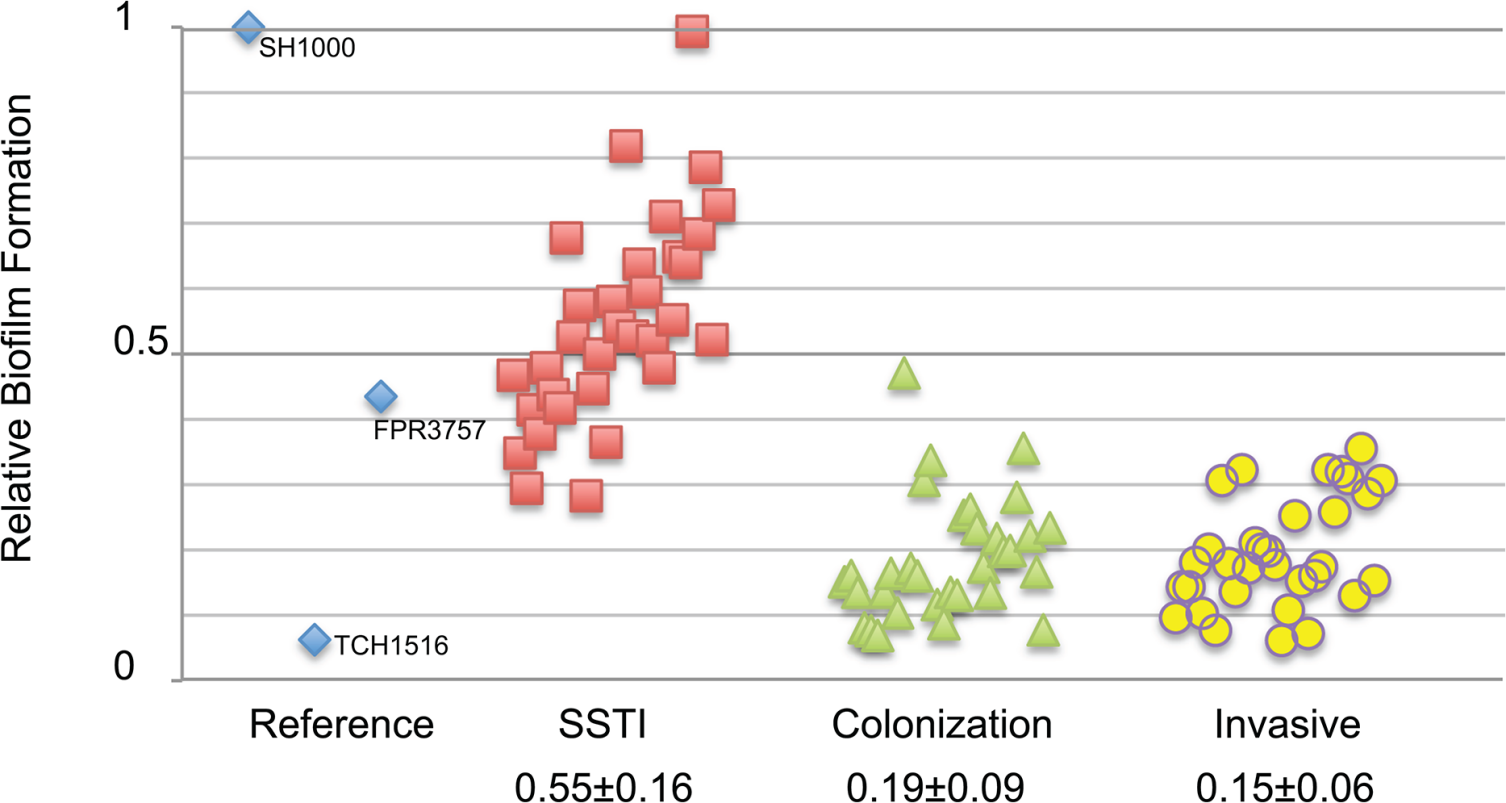
Correlation of biofilm forming potential and SSTI isolates of CA-MRSA clinical isolates. Biofilm formation was assessed using microtiter assay as described previously for 96 clinical isolates of *S*. *aureus* representing 3 different types of host-pathogen interactions. Distribution of data is shown by scatter plot. Each data point is representative of the mean OD_600_ of 9 replicates of each individual isolate relative to the SH1000 reference strain. Relative biofilm formation mean ± SD for each host-pathogen interaction is shown.

Supporting the idea that environmental surfaces contribute to the transmission of CA-MRSA [31,45–47], we did observe a small, but significant, correlation between biofilm potential and SSTIs infections (Fig 6). SSTI strains (0.55 ± 0.16) had higher relative biofilm formation than colonizing strains (0.19 ± 0.09) and invasive strains (0.15 ± 0.06; p<0.001, Table 2).

## Discussion

Several conclusions can be drawn from this study. First and foremost, our finding that domesticated *S*. *aureus* strains exhibit a high degree of phenotypic variation highlights the well known problems associated with repeated passaging and mutagenesis of bacterial stocks [48–53]. Significantly, although all the domesticated strains are virulent in mouse models, several of the phenotypes that exhibited the highest degree of variation have been implicated in *S*. *aureus* virulence, including biofilm formation and hemolysis. In contrast to the laboratory strains, minimally passaged clinical isolates exhibited only modest phenotypic variation regardless of origin. The latter finding is consistent with data from other laboratories suggesting that *S*. *aureus* is under a high degree of selective pressure in the “wild”[54]. Below we discuss the implications of this work.

Phenotypic analysis of the nine domesticated *S*. *aureus* strains is consistent with a high degree of genetic drift, leading to differential regulation of *agr* [3,35,55–57]. This is not surprising for older strains such as NCTC8325 and Newman, which were originally isolated 71 and 62 years ago respectively, but is somewhat unexpected in more recent isolates such as TCH1516 and FPR3757. In particular, genetic differences in hemolytic capacity and biofilm formation are likely related to *agr* status of individual strains [58,59]. *agr* positive strains often exhibit strong hemolysis and poor biofilm formation and *agr* negative strains, exhibit poor hemolysis and a strong propensity for biofilm formation. Like many non-virulent bacterial models including *E*. *coli* K12 derivatives, several of these strains have been subjected to mutagenesis in the course of their natural history to make them more tractable. SH1000, a man made strain commonly used for biofilm studies, is itself a derivative of NCTC8325 that was subjected to mutagenesis to cure prophage and subsequent repair of *rsbU* [9,10]. It is perhaps thus not surprising that SH1000 and its parent strain NC8325 are phenotypically divergent with regard not only to colony pigment, but also sensitivity to autolysis, biofilm forming potential, and hemolysis of sheep’s blood (Figs 1, 2, 3 and 4).

At the same time, the high degree of variation we observed in phenotypes implicated in virulence, particularly biofilm formation and hemolysis, is surprising as all of the domesticated strains, with the exception of NCTC8325 and Mu50, are employed more or less interchangeably for studies of virulence determinants in mouse models. Our findings thus highlight not only the robust nature of *S*. *aureus* pathogenesis and the depth of its virulence “arsenal”, but also the need for caution when assessing the significance of individual virulence determinants in *S*. *aureus* infection.

Although phenotypic observations revealed significant variation amongst all the strains, the CA-MRSA strain TCH1516 emerged as an outlier. We speculate that TCH1516 lost the ability to synthesize heme in the heme rich environment of the host circulatory system, and thus acquired this “hyper-hemolytic” ability as a means to compensate. Closer examination of the sequence of TCH1516 revealed several mutations that may contribute to the strain’s unique phenotype. One such mutation is in *hemE*, a precursor for heme biosynthesis in *S*. *aureus* [17]. Defects in heme biosynthesis, similar to defects in menaquinone biosynthesis, have been associated with the small colony variant (SCV) phenotype [60–63].

Another possible contributor to the peculiar phenotype of USA300 TCH1516 is the mutation in an AraC family transcriptional regulatory (AFTR) protein [17]. Proteins in this family are regulators of carbon metabolism, stress response, and virulence that respond to changing environmental conditions [64–66]. Mutations in AFTRs have previously been associated with the transition of a colonizing strain of *S*. *aureus* to one that caused an invasive septic infection [67]. Further examination of this class of proteins will be necessary to pinpoint a specific role in *S*. *aureus* metabolic adaptation to the bloodstream.

In contrast to the domesticated strains, we observed little phenotypic variation between the 300 minimally passaged clinical isolates. At the same time, we did observe small but significant correlations between host-pathogen interaction and three of the four phenotypes tested. These included a modest but statistically significant increase in the frequency of invasive isolates with defects in pigment formation and acetate utilization, as well as tendency of SSTI isolates to exhibit slightly higher levels of biofilm formation (Table 2). While the underlying cause of the former is unclear, the latter correlation is consistent with the idea that these types of infections are biased towards those bacteria best able to “stick” to abiotic surfaces. The ability of the CA-MRSA clinical isolates to attach to inert surfaces promotes transmission and enhances their capacity to cause further infections [31,45]. At the same time, the correlation was modest. Being a low “performer” with regard to biofilm forming ability is not necessarily a barrier to entry so to speak when it comes to initiating an SSTI.

Phenotypic uniformity among the clinical isolates is consistent with a high degree of evolutionary pressure on *S*. *aureus* from both host and environmental factors. For example, colonization of the epidermis is dependent upon evasion of host-specific cutaneous defenses mediated by toll-like receptors [31,68]. *S*. *aureus* must also contend with the presence of commensal bacteria such as *Staphylococcus epidermidis* on the host. *S*. *epidermidis* has also been shown to directly inhibit S. aureus colonization in preferred environmental niches [31,68].

The high degree of phenotypic uniformity between clinical isolates regardless of origin, suggests that stochastic events and host-specific factors are more important determinants of the host-pathogen relationship than variations between individual *S*. *aureus* strains. This idea is not new. *Ruimy et al*. reported that host-associated factors are the predominant determinant of *S*. *aureus* nasal carriage [54]. Similarly, a 15 month evaluation of the genome sequence of persisting *S*. *aureus* strains by *Uhlemann et al*, revealed the accumulation of mutations in genes involved in all aspects of host colonization [45]. This observation of variance in the *S*. *aureus* genome over time is consistent with the idea that host biology is a critical determinant of the *S*. *aureus* host-pathogen relationship. We anticipate that future work investigating the contribution of host factors to *S*. *aureus* pathogenesis will strengthen our knowledge of virulence factor evolution as well as give insights to the transmission and spread of staphylococcal disease.

## Materials and Methods

### Strains & Growth Conditions

All *S*. *aureus* strains are listed in Table 1. *S*. *aureus* was cultured in Tryptic Soy broth (TSB) medium unless otherwise noted and cells were grown at 37°C. Unless otherwise stated cultures are grown from a single colony until mid-log (OD_600_ of ~0.2 – 0.6), then diluted to an OD_600_ of ~0.05 and grown to mid-log again and used for further study.

With regard to the clinical strains, wound isolates were sampled directly from the source of infection and plated directly to blood agar. Single colonies were sub-cultured once on solid medium for purity and again for antibiotic susceptibility prior to freezing. Colonization isolates (nose, armpit, groin), were subjected first to a broth enrichment step in Trypiticase soy broth with 6.5% NaCl prior to colony purification and testing for antibiotic susceptibility

### Dilution Plating

*S*. *aureus* strains USA300 TCH1516 and USA300 FPR3757 were dilution plated similarly to previous description [69]. Cells were grown to stationary phase in TSB medium, and then diluted back to an OD_600_ of 0.05 upon which serial dilutions of 10^−1^ to 10^−5^ were made into fresh TSB medium. 5ul of each dilution was then plated in series onto either pre-warmed Tryptic Soy Agar (TSA), TSA II plates + 5% sheep’s blood (Fisher), or TSA plates supplemented with Human hemoglobin (Hb) (Sigma). Liquid cultures were allowed to dry on plates for up to an hour, and then plates were incubated at 37°C overnight. The plates were then imaged and the relative growth at each media condition was qualitatively assessed.

### Hemolysis Plating

Single colonies of *S*. *aureus* are inoculated in triplicate to 5ml of TSB media. Cultures are grown with shaking at 37°C to mid-log (OD_600_ 0.2–0.6). Cultures are then diluted to an OD_600_ of 0.05 in a microcentrifuge tube. 5ul of diluted cultures are spotted onto TSA plates with 5% sheep’s blood agar [69] and incubated at 37°C for 48hrs. After 48hrs zones of hemolysis are observed and measured. Plate images are scanned and saved.

### Biofilm Assay

*S*. *aureus* is grown from single colonies to mid log (OD_600_ 0.2 – 0.6) in 5mls of TSB media. Culture is diluted to OD_600_ 0.05 in TSB media + 0.5% glucose. 200ul of each culture is then aliquoted into 96 well polystyrene plates. 96 well plate is covered and incubated statically at 37°C for 24hrs. After 24hr period media with non-attached cells is discarded. 96 well plates are then washed 3 times with de-ionized water. Plates are air-dried for at least 15 minutes. 200ul of ethanol is added to each well for 1 min, and then removed using pipet. Plates are then allowed to air dry for at least 5 minutes. All wells are then stained with 200ul of 0.5% crystal violet for 10 minutes at room temperature. After 10-minute incubation period crystal violet is discarded and 96 well plates are washed 3 times with DI water. All wells are washed with 33% acetic acid, and then diluted 1:10 in distilled water. OD_595_ is then measured using a spectrophotometer.

### Autolyis Assay

Strains of *S*. *aureus* are streaked from ice onto TSA plates + 5% sheep’s blood and grown overnight at 37°C. Single colonies are picked for each strain and grown in 5ml TSA media with aeration until stationary phase (OD600 > 1.5). Cultures are then backdiluted to an OD600 of ~0.05 in 2ml of TSA media and grown for 3–5 hours at 37°C with aeration to mid-exponential phase (OD600 ~0.7–1.0). Following 3–5 hours of growth, cultures are quickly chilled on ice for 10 minutes and 1ml aliquots are harvested by centrifugation (3,200 x g for 5min at 4C). Supernatant is discarded and remaining pellet is washed with ice-cold water once and then re-suspended in autolysis buffer (0.5M Tris-HCl pH 7.5, 0.05% TX-100) [26,27]. 100ul of each strain is then added to 96 well microtiter plates in triplicate. *S*. *aureus* strains are grown at 37°C with aeration in microtiter plates using BioTek Eon. OD_600_ readings are taken every 30min.

### Screen for loss of acetate metabolism

Strains of *S*. *aureus* are streaked from ice onto TSA plates + 5% sheep’s blood and grown overnight at 37°C. Single colonies are picked for each strain and grown in 2ml TSA media containing 1ml/L of a 1% solution of Phenol red. Ratio of growth container to culture medium ration should be at least 10:1. Culture tubes are incubated at a 45-degree angle to allow for maximum aeration, and shaken at 225rpm. *S*. *aureus* strains are grown overnight (12 – 18 hours) at 37°C. The media is observed for color changes after overnight growth. Yellow cultures are indicative of an acidic pH. Experiment was done in duplicate on three separate days for each strain.

### Statistical analysis

Data were analyzed using SPSS 22 for Windows (IBM SPSS, Chicago, IL). Phenotypic differences between the three categories of clinical *S*. *aureus* isolates (colonization, SSTI, and invasive infection) were analyzed using the Pearson's χ^2^ test. All tests for significance were 2-tailed, and p-values of <0.05 were considered significant.
